# Supplementary material for: Inconsistent PCR detection of Shiga toxin-producing Escherichia coli: Insights from whole genome sequence analyses
Source: PLoS One. 2021 Sep 3;16(9):e0257168. doi: 10.1371/journal.pone.0257168 (PMC8415614; doi:10.1371/journal.pone.0257168)
Supplement: S1 Table — (DOCX) [file pone.0257168.s001.docx]

**SUPLEMENTARY FILE**

**S1 Table.** Virulence genes detected in all STEC *E. coli* sequences.

| Gene | Action/Regulation | Reference |
| --- | --- | --- |
| *cheY* | Chemotaxis regulator protein *CheY* | NCBI |
| *csgB* | Chemotaxis regulator protein *CheY* | NCBI |
| *csgD* | "curli subunit gene D," is a transcriptional regulator that regulates a number of genes involved in the curli assembly, transport, and structural components, which are important for biofilm formation | NCBI |
| *csgE* | May be involved in the biogenesis of curli organelles. | UNIPROT |
| *csgF* | *csgF* when overexpressed causes abnormal biofilm architecture | NCBI |
| *csgG* | *csgG* when overexpressed causes abnormal biofilm architecture. *csgG* is a lipoprotein located in the outer membrane. | NCBI |
| *entA* | An intermediate in the enterobactin biosynthesis pathway. | NCBI |
| *entB* | Enterobactin synthase component B | UNIPROT |
| *entC* | There are two isochorismate synthase enzymes present in *Escherichia coli*, encoded by entC and menF. | NCBI |
| *entE* | This gene is involved in the pathway enterobactin biosynthesis | UNIPROT |
| *entF* | Apo-serine activating enzyme is phosphopantetheinylated posttranslationally resulting in the active enzyme form, serine activating enzyme. | NCBI |
| *entS* | *entS* protein is a member of the major facilitator superfamily (MFS) of transporters. | NCBI |
| *espL1* | Putative type III secreted effector | UNIPROT |
| *espX1* | Putative type III secreted effector | UNIPROT |
| *espX4* | T3SS effector-like protein *EspX* | UNIPROT |
| *espX5* | effector protein translocated by the type III secretion system | Bugarel et al. 2011 |
| *fdeC* | Intimin-like adhesin | UNIPROT |
| *fepA* | *fepA* is a protein involved with transport of enterobactin-iron across the outer membrane | NCBI |
| *fepB* | *fepBCDG* are components of a ferric enterobactin transport complex that is a member of the ATP-binding cassette (ABC) family of transporters. | NCBI |
| *fepC* |  |  |
| *fepD* |  |  |
| *fepG* |  |  |
| *fes* | Enterochelin esterase | UNIPROT |
| *fimA* | Type-1 fimbrial protein, A chain | UNIPROT |
| *fimB* | *fimB*, along with *fimE*, is a recombinase in *Escherichia coli.* The *fim* switch (*fimS*), to control transcription of the type I fimbrial structural genes--a process known as phase-variation switching. | NCBI |
| *fimC* | *fimC* is a member of the periplasmic chaperone family which functions in the chaperone-usher pathway and is indispensable in the biogenesis of the type 1 pilus fiber of *Escherichia coli*. | NCBI |
| *fimD* | *fimD* forms pores in the outer membrane. *fimD* is a member of the Fimbrial Usher Porin (FUP) family. | NCBI |
| *fimF* | Involved in regulation of length and mediation of adhesion of type 1 fimbriae | UNIPROT |
| *fimG* | Involved in regulation of length and mediation of adhesion of type 1 fimbriae (but not necessary for the production of fimbriae) | UNIPROT |
| *fimH* | Involved in regulation of length and mediation of adhesion of type 1 fimbriae (but not necessary for the production of fimbriae). Adhesin responsible for the binding to D-mannose | UNIPROT |
| *fimI* | *fimI* gene is located in the *fim* gene cluster of *Escherichia coli* and has been shown to encode a 16.4 kDa noncytoplasmic protein product. | NCBI |
| *flgG* | *flgG* is one of four proteins that comprise the rod section of the basal-body assembly of the flagellar motor | NCBI |
| *fliM* | *fliGMN* switch complex on the rotor of the bacterial flagellum. *fliM* is one of three components of the flagellar motor's "switch complex | NCBI |
| *ompA* | *ompA* is required for colicins K and L and the stabilization of mating aggregates | NCBI |
| *stx1A and B* | Shiga toxin 1 a and b-subunit | UNIPROT |
| *yagV/ecpE* | Probable fimbrial chaperone *ecpE* | UNIPROT |
| *yagW/ecpD* | fimbrial adhesin EcpD. YagW is a hypothetical protein. | NCBI |
| *yagX/ecpC* | Putative fimbrial usher protein *ecpC*. Contains PPE repeats. | NCBI |
| *yagY/ecpB* | Putative fimbrial chaperone. O18K1H7 matABC genes are required for Mat fimbria formation when cloned in K-12. MatC is associated with the expression of Mat fimbriae in the meningitis-causing *Escherichia coli* O18K1H7 | NCBI |
| *yagZ/ecpA* | Common pilus major subunit. | NCBI |
| *ykgK/ecpR* | DNA-binding transcriptional dual regulator *MatA* | NCBI |
